# Supplementary material for: Patterns of Intron Gain and Loss in Fungi
Source: PLoS Biol. 2004 Nov 30;2(12):e422. doi: 10.1371/journal.pbio.0020422 (PMC532390; doi:10.1371/journal.pbio.0020422)
Supplement: Table S1 — Also available at http://genes.mit.edu/NielsenEtAl/. (4.3 MB ZIP). [file pbio.0020422.st001.zip › NielsenEtAl/html/1203.html]

AN6111.1.NCU00334.1.MG08860.1.FG05509.1


```
 CLUSTAL W (1.82) Multiple Sequence Alignments - Introns Inserted


Sequence 1: NCU00334.1	297 aa
Sequence 2: FG05509.1	293 aa
Sequence 3: MG08860.1	251 aa
Sequence 4: AN6111.1	295 aa
Alignment Length: 298 aa
Number Identitical Residues: 90 aa
Alignment Score (without introns) 5898


MG08860.1 	MPREAEPSLNEKAFLLQALQ-QNLRLDGRDIDEYRPIKLSFGDEYGLVEVEFGKTR2IVA
NCU00334.1	MPREAEPSANEKAFLLKALQDEQIRLDGREFDQYRPLELTFGDEYGVANVSLGKTK2ILA
FG05509.1 	MPREAEPSLSEKTFLAQALN-EGLRLDGRKFDEFRPLEITFGDEYGVAEVKYGKTR2VLA
AN6111.1  	MNKEAPLSIAERDFILDALR-EGVRLDGRGADQLRPLTLSFGEEYGHVKVQLGKTS2LVV
          	* :**  *  *: *: .**. : :*****  *: **: ::**:*** .:*. ***  ::.

MG08860.1 	KASAEVTIPYTDRPFDGIFNIVTELSPMASPAFEVNR~PTETEVLLSRLLEKTIRRSNAL
NCU00334.1	KASAEVTVPFADRPLDGIFTIATELSPMAAPHFEVNR~PTETEVLLSRLLEKTVRRSGAL
FG05509.1 	KVSAEVTVPYTDRPFDGVFTITSELSPMVAPSYEVNR~PSLEEVLLSRLLEKTIRRSGAL
AN6111.1  	RISAEVAKPHDDRPFDGIFNIAMELTAMGSPAWENGR2QGDLETYVTNVLDRVIRHSNAL
          	: ****: *. ***:**:*.*. **:.* :* :* .*     *. ::.:*::.:*:*.**

MG08860.1 	DTESLCLVAGQKCWSIRVDVHAESHDGNLIDASCLAVVAALRHFRKPDTSIEGEVVTVYT
NCU00334.1	DTESLCLVAGQKCWSVRVDVHVLSHDGNLTDAACFAVVAALRHFRKPDTSMENGVLTVYT
FG05509.1 	DTESLCLIAGKKCWSIRVDLHVLAHDGNLTDAACVAVIAALRHFRKPDSSIEGETLTIYT
AN6111.1  	DTESLCILKGVSCWTIRADVHITDYDGNLIDASCIGVMAGLQHFRRPDAVVKDGQVIVYG
          	******:: * .**::*.*:*   :**** **:*..*:*.*:***:**: ::.  : :* 

MG08860.1 	PAEREPVPLSWLHSPFCVTFSFFGDLGDMFVVDANWLEEQVH1RKARRDRRRSRHTAAVQ
NCU00334.1	PAEREPVPLGWLHSPFCVTWSFFGEEGETALLDADWMEEQVR~-VASVTISLNRHGEICQ
FG05509.1 	PAEREPVPLSWLHSPFCVTYSFYGEDGSQVLVDTNWLEEKMR~-VSHCTYSLNKHGEISQ
AN6111.1  	LDERVPAPLNITHKPLSVTFHTFDD-GKLAILDATRKEEQAS~-EADVVIGMNNAGDVCY
          	  ** *.**.  *.*:.**:  :.: *.  ::*:   **:     :      ..      

MG08860.1 	RGGVG----QGQGFLQAPR-----------------------------------------
NCU00334.1	IAKLGGVPVEAVALLQCTSVALTKVKEFSTYLDDKLAEDAKRRDKGGFMAELRAENDRIV
FG05509.1 	ISKLGGKALDAPLFIQCAQGALNRSKDLSNIVDNKLAEDAKRRDKGGLMAELTAENDR--
AN6111.1  	LSKFQGSPVDALVFITKTSLALEKVKYINGIIDKALQADLAKRSKGGLAEQARAENDRPV
          	 . . . . :.  ::  .  :    .  .   ..    .    ....   .  :...   

MG08860.1 	-
NCU00334.1	G
FG05509.1 	-
AN6111.1  	A
          	.
```
